# Cell Cycle-Dependent Recruitment of FtsN to the Divisome in Escherichia coli

**DOI:** 10.1128/mbio.02017-22

**Published:** 2022-08-15

**Authors:** Jaana Männik, Sebastien Pichoff, Joe Lutkenhaus, Jaan Männik

**Affiliations:** a Department of Physics and Astronomy, University of Tennessee, Knoxville, Tennessee, USA; b Department of Microbiology, Molecular Genetics, and Immunology, University of Kansas Medical Center, Kansas City, Kansas, USA; University of Nebraska Medical Center

**Keywords:** *Escherichia coli*, FtsA, FtsN, bacterial cell division, cell cycle checkpoints, constriction formation, fluorescence live-single-cell microscopy, microfluidics

## Abstract

Cell division in Escherichia coli starts with the formation of an FtsZ protofilament network at midcell, the Z ring. However, only after a considerable lag period does the cell start to form a midcell constriction. The onset of constriction depends upon the arrival of so-called late divisome proteins, among which, FtsN is the last essential one. The timing and dependency of FtsN arrival to the divisome, along with genetic evidence, suggests it triggers cell division. In this study, we used high-throughput fluorescence microscopy to determine the arrival of FtsN and the early divisome protein ZapA to midcell at a single-cell level during the cell cycle. Our data show while the recruitment of ZapA/FtsZ is gradual in the cell cycle, recruitment of FtsN is rapid and begins at about the onset of constriction. At this time, the fraction of ZapA/FtsZ in the Z ring approaches its peak value. We also find a second increase in FtsN recruitment to the divisome, which begins once the amount of ZapA/FtsZ at midcell starts decreasing. Increasing hypermorphic FtsA* (FtsA R286W), but not FtsA, accelerates FtsN recruitment but not constriction. This finding is consistent with FtsA* recruiting FtsN with some other divisome component being rate-limiting for constriction under these conditions. Finally, our data support the recently proposed idea that ZapA/FtsZ and FtsN are part of physically separate complexes in midcell throughout the whole septation process.

## INTRODUCTION

Cell division in bacteria requires the regulated assembly of several dozen divisome proteins that collectively initiate and guide the formation of the constriction/septum, which divides the mother cell into two daughters (1, 2). In Escherichia coli, the assembly of the essential divisome proteins involves at least two stages (3, 4). During the first stage, the tubulin-like GTPase FtsZ polymerizes into dynamic protofilament assemblies (5) that are attached to the inner leaflet of the cytoplasmic membrane by the membrane tethers FtsA and ZipA (6). These protofilament assemblies (Z ring) are topologically restricted by factors affecting FtsZ polymerization so that the Z ring forms at midcell (1). The assemblies are decorated by the nonessential Z ring associated protein, ZapA (3, 5, 7), and potentially with other less conserved and less abundant Zap proteins (ZapC, ZapD) (8–10). This initial complex forms a highly dynamic discontinuous ring-like structure, referred to as the Z ring, composed of FtsZ protofilaments that treadmill around the division plane (11–13). FtsEX also arrives at this stage and is required for the recruitment of proteins during the second stage (14, 15).

During the second stage, the remaining essential divisome proteins, including the enzymes necessary to synthesize septal peptidoglycan (PG), are recruited to the midcell to complete the assembly of the mature divisome, which starts to synthesize the septal cell wall (1, 2). Dependency studies indicate sequential recruitment of these late divisome proteins with the order FtsK<FtsQ<FtsB-FtsL<FtsW-FtsI<FtsN (1, 2). FtsN is the last of the essential proteins to arrive, and it has been postulated to be the trigger protein that leads to the onset of constriction (16–21). FtsN is an integral membrane protein (22) with a short cytoplasmic domain (FtsN^cyto^), a single transmembrane helix followed by a large periplasmic domain (23). The periplasmic domain is further divided into two subdomains: an essential domain (FtsN^E^), which has been implicated in triggering septal PG synthesis (17, 24), and a C-terminal SPOR (septal peptidoglycan binding) domain, which interacts with PG strands denuded of peptide linkages by amidases (16, 25, 26).

The recruitment of FtsN to midcell is complex. It has been reported the cytosolic domain of FtsN (FtsN^cyto^) can be recruited to the division site via its binding to FtsA (27–30). However, for the recruitment of full-length FstN, FtsQ, FtsI, and all upstream proteins are likely required (1, 31, 32). FtsN, along with ZipA, is proposed to interact with the PG synthases PBP1a and PBP1b to initiate a preseptal phase of PG synthesis (33). FtsN is also recruited via its SPOR domain that binds to denuded glycan strands produced by activated amidases (16, 34). Because the denuded strands are formed following the onset of septal cell wall synthesis, the recruitment process of FtsN via its SPOR domain is thought to be self-enhancing (also referred to as the septal PG loop) once constriction starts (16, 22, 25).

Current models on how FtsN initiates the onset of constriction envision a two-pronged mechanism (17, 35–37). In these models, FtsN^E^ causes a conformational change in FtsQLB in the periplasm that activates FtsWI (36). A parallel activation pathway in the cytoplasm involves FtsN^cyto^ activating FtsA (17, 28–30). The FtsA mutant R286W (38), with reduced self-interaction, is thought to mimic an active state of FtsA and will be denoted here as FtsA*. FtsA* acts directly on FtsW in the cytoplasm (39). Under physiological conditions these pathways synergize to activate FtsWI. However, mutations in *ftsL* or *ftsB* that hyperactivate the periplasmic pathway or in *ftsA* that hyperactivate the cytoplasmic pathway are capable of leading to cell constriction in the absence of the other (1, 17, 35).

While FtsN is essential for cell division and potentially a trigger for it, the kinetics of its recruitment to the divisome has not yet been determined. All existing data on FtsN recruitment to the divisome originates from static cell studies or imaging studies where short time-lapse series have been taken. Here, we study FtsN dynamics at the individual cell level throughout the cell cycle and determine its recruitment kinetics to the divisome. Our high-throughput studies show that while the recruitment of ZapA/FtsZ to midcell is gradual, recruitment of FtsN is abrupt and occurs on average about a quarter of the cell cycle after a persistent Z ring forms. We find at the time of FtsN recruitment, the fraction of ZapA/FtsZ in the Z ring reaches its peak value. Furthermore, our data show the recruitment of FtsN to midcell occurs in two distinct stages. During the first stage constriction initiates, but the speed of septal closure increases in the second stage, which starts when ZapA/FtsZ numbers at the midcell start to decrease. The presence of FtsA* accelerates FtsN recruitment but does not lead to earlier constrictions. Our data also supports the idea FtsN and ZapA/FtsZ are part of spatially separate complexes throughout the division process.

## RESULTS

### FtsN accumulates rapidly at midcell about a quarter of the cell cycle time after the formation of a persistent midcell ZapA/Z ring.

To investigate the recruitment kinetics of FtsN to the divisome in live cells, we used quantitative fluorescence microscopy of a functional N-terminal fusion of Ypet to FtsN (Ypet-FtsN) (40). We studied the kinetics of FtsN relative to the formation of the Z ring. As a proxy for the Z ring, we used a functional C-terminal fusion of mCherry to ZapA (ZapA-mCherry) (34). Both fusions are expressed from the native chromosomal locus. ZapA is a highly conserved, although not essential early cell division protein that binds to FtsZ (41). We found the ZapA fraction at midcell is precisely the same as the FtsZ fraction at midcell throughout the cell cycle (Fig. S1A) and the width of their midcell accumulations is also the same (Fig. S1B, for details of midcell intensity calculation, see Fig. S1C and D and Text S1). The same partitioning ratio of FtsZ and ZapA at midcell indicates they bind to each other at a fixed stoichiometric ratio irrespective of whether FtsZ protofilaments are present as transient assemblies or are part of a mature divisome that synthesizes septal PG. A fixed stoichiometry between FtsZ and ZapA has also been observed *in vitro* studies (42). Altogether, these data indicate ZapA-mCherry acts as a faithful reporter for FtsZ.

10.1128/mbio.02017-22.1 FIG S1 ZapA-mCherry is a faithful reporter for FtsZ and schematic drawing for the calculation of the midcell intensity (A) The excess fraction of ZapA (red) and FtsZ (blue) at midcell, and (B) the width of their midcell accumulation as a function of cell age for slow-growing cells (glycerol-TrE media, *N* = 63). FtsZ-GFP in the strain is expressed from a plasmid at about 40% level of the native protein (13). ZapA-mCherry is expressed from its native locus. A total of 30 μg/mL chloramphenicol is used in these measurements. The width of the midcell accumulation is determined based on a Gaussian fit of the line profile from the ZapA-mCherry or Ypet-FtsN fluorescent signal (see Text S1). (C) Schematics showing how fluorescence intensity line profiles along the long axes of the cell were calculated. For further details of the procedure, see Text S1, *Image analysis*. (D) Schematics showing how excess fraction of the signal (% of midcell) was calculated. See Text S1, *Compilation of midcell intensity versus time traces* for further detail*s*. Download FIG S1, EPS file, 1.4 MB. Copyright © 2022 Männik et al. 2022 Männik et al. https://creativecommons.org/licenses/by/4.0/ This content is distributed under the terms of the Creative Commons Attribution 4.0 International license.

**FIG 1:**
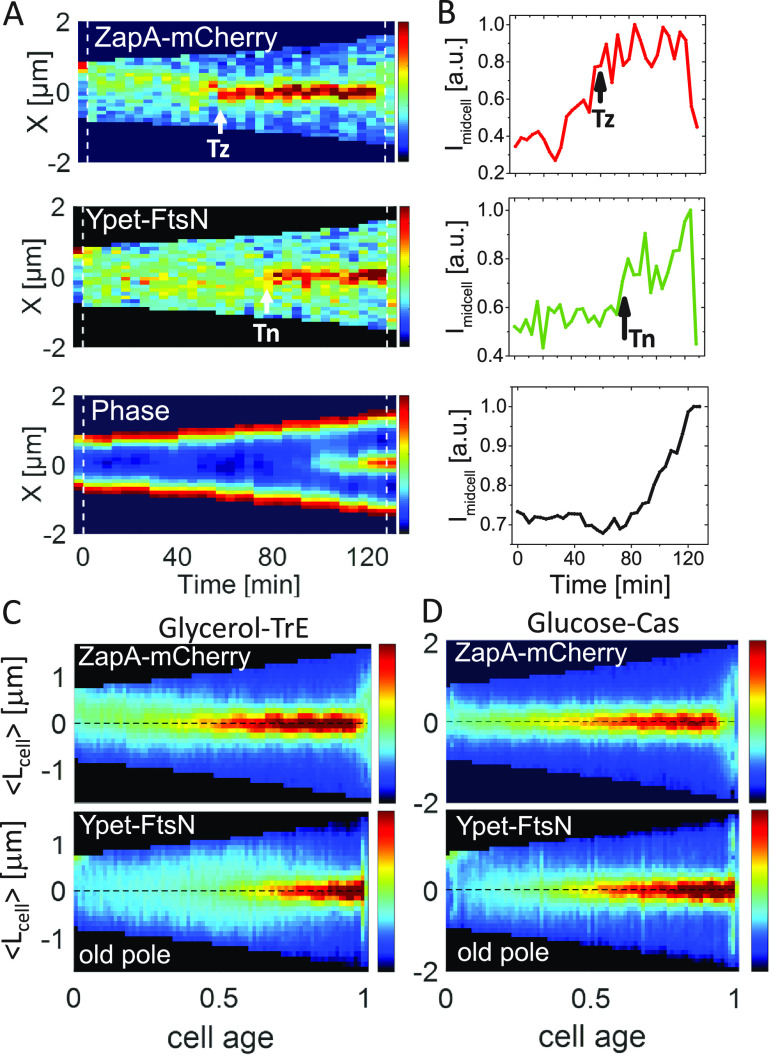
Accumulation of ZapA and FtsN at midcell. (A) Kymographs of fluorescent and phase signals for a representative cell grown in M9 glycerol-TrE medium. Red corresponds to high- and blue to low-intensity values. Black marks regions outside the cell. Dashed vertical lines indicate cell division events. The arrows indicate event timings as determined by an automated algorithm (see Text S1). The timing of the persistent Z ring is denoted by *Tz* and the onset of FtsN recruitment by *Tn*. (B) Midcell intensity traces of ZapA-mCherry (top), Ypet-FtsN (middle), and phase signal (bottom) for the cell shown in the kymograph. The intensity traces were collected from about a 0.5 μm wide band in the cell middle. No cell background was subtracted from these traces. a.u., arbitrary unit. (C and D) Population averaged kymographs of ZapA-mCherry and Ypet-FtsN signals in M9 glycerol-TrE (*N* = 339) and glucose-cas (*N* = 526) media, respectively. All cells are aligned so that their old pole is at the bottom of the graph. The dashed horizontal line shows the midline of the cell. For further details, see Text S1.

10.1128/mbio.02017-22.10 Text S1 Contains details on: (i) cell preparation and culture in microfluidic devices; (ii) fluorescence microscopy; (iii) image analysis; (iv) compilation of mid-cell intensity versus time traces; (v) determination of *Tn* and *Tz* timings, and width of accumulations; (vi) compilation of cell age plots in Fig. 1 and (vii) Western blot analysis. Download Text S1, DOCX file, 0.04 MB. Copyright © 2022 Männik et al. 2022 Männik et al. https://creativecommons.org/licenses/by/4.0/ This content is distributed under the terms of the Creative Commons Attribution 4.0 International license.

Although a sole copy of Ypet-FtsN was expressed from its endogenous locus, a Western blot of our FtsN construct shows in addition to full-length Ypet-FtsN, there is also a truncated form present (Fig. S2A). Such fragments have also been observed in other studies where N-terminal fusions to FtsN have been used (43, 44). The combined abundance of these two proteins is approximately the same as that of FtsN in the wild-type (WT) strain. Our anti-GFP antibodies did not bind any of the bands on the Western blot. However, in an earlier study, the fragments were found to correspond to the degradation of the N-terminal fluorescent fusion protein (44). There were no degradation bands in WT cells where only a native FtsN was present. The latter finding is also consistent with the notion that degradation affected Ypet rather than FtsN.

10.1128/mbio.02017-22.2 FIG S2 Western blot to determine (A) native FtsN and Ypet-FtsN amounts and (B) FtsA amounts after overexpression from plasmid pSEB306+ in cells grown in M9 media supplemented with glucose-cas. (A) Left, Western blot image for FtsN detection: lane 1, Odyssey protein marker, lanes 2 to 4, BW27783 cells with native FtsN; lanes 5 to 7, JM144 cells expressing a Ypet-FtsN fusion. Anti-FtsN serum was used at a final concentration 1:350 (UK43, Lutkenhaus lab). The strain with Ypet-FtsN shows a full-length construct for Ypet-FtsN (fusion) and a truncated construct (fragment) but no native FtsN band. Right, Histogram showing normalized FtsN amounts from the Western blot quantified using ImageJ software. For correction band between 25 and 37 kDa is used; otherwise, the procedure follows that for FtsA. The average of three samples is shown. The error bars correspond to SD. (B) Left, Western blot image for FtsA detection: M, Odyssey protein marker (cat#928-40000), lane 1 to 3, JM150 (pDSW210-FtsA) cells without induction; lane 4 to 6, JM150 cells induced with 100μM of IPTG; lane 7 to 9, JM149 (pDSW210) cells induced with 100μM of IPTG. anti-FtsA serum was used at a final concentration 1:750 (UK54, Lutkenhaus lab). The secondary donkey anti-rabbit IRDye 680RD antibodies were used at the dilution of 1:15,000. Images of the near-infrared fluorescent signal were acquired using an Odyssey scanner (LI-COR Biosciences) with details in the Material and Methods section of the online supplemental materials. The topmost of the two close bands corresponds to FtsA based on its upregulation during IPTG induction of plasmid pSEB306+. Right, Histogram showing normalized FtsA amounts of Western blot quantified using ImageJ software. The average of three samples is shown. The integrated intensity in the topmost band is used for FtsA. This intensity is corrected by dividing it by the integrated intensity in the bottommost band. Both integrated intensities are found by fitting the intensity line profiles from each lane by two Gaussians in Origin Pro 2016. After averaging over three bands, the data from different strains is further normalized, using the value for the strain with an empty vector (JM149). The error bars correspond to SD. Download FIG S2, EPS file, 0.3 MB. Copyright © 2022 Männik et al. 2022 Männik et al. https://creativecommons.org/licenses/by/4.0/ This content is distributed under the terms of the Creative Commons Attribution 4.0 International license.

We imaged cells carrying ZapA-mCherry and Ypet-FtsN in mother-machine microfluidic devices under moderately fast- and slow-growth conditions. Cells grew in these devices in steady-state conditions with doubling times of *Td* = 78 ± 28 min (mean ± SD) and *Td* = 143 ± 45 min, respectively. Note all measurements were carried out at 28°C, where the growth rate is expected to be about one-half of that at 37°C (45). These doubling times and cell lengths were comparable to the parent strain without the Ypet-FtsN and ZapA-mCherry labels indicating the fluorescent tags did not affect cell parameters (Table S1) even though Ypet in our Ypet-FtsN can weakly dimerize and potentially interfere with the interaction of FtsN with FtsA. The measurements where FtsA* is upregulated (as described later) also argue against the possibility that FtsN interaction with FtsA is significantly impacted. At the single-cell level, the midcell accumulation of ZapA at the slow-growth rate increased gradually prior to the formation of a persistent Z ring (Fig. 1A and B, top; for representative cell images, see Fig. S3A). This finding is consistent with the previous reports which monitored FtsZ (5, 46). The overall increase in the ZapA amount at midcell was interspersed by large fluctuations in ZapA numbers in the first half of the cell cycle, reflecting the appearance and disappearance of FtsZ transient assemblies (5). In the majority of cells, weak FtsN foci could also be seen in the first half of the cell cycle. These foci did not colocalize with the ZapA-mCherry assemblies (cf. Fig. 1A, top and middle panels). Inspecting the whole-cell population revealed ZapA-mCherry transient assemblies formed preferentially at the new pole half of the cell in slow-growth conditions (Fig. 1C, top). At the same time, the Ypet-FtsN ones were distributed evenly between cell halves so that the peak in their distribution occurred at midcell (Fig. 1C, bottom). Accumulation of FtsZ transient foci in the new pole half of the cell has previously been explained by the Ter linkage mechanism (47). In moderately fast-growth conditions (glucose-cas medium), the period when transient foci could be observed was significantly shorter, and the transient ZapA/FtsZ assemblies did not preferentially localize to the new pole half of the cell (Fig. 1D). An inspection of the single cell data, such as in Fig. S3B to D, also showed that Ypet-FtsN and ZapA-mCherry foci did not colocalize in the early cell cycle. Overall, these data indicate Ypet-FtsN is not recruited to ZapA/FtsZ transient assemblies.

10.1128/mbio.02017-22.3 FIG S3 Representative images of studied cells and accumulation of ZapA and FtsN at midcell in moderately fast-growing cells in glucose-cas medium. (A and B) Fluorescent and phase images of cells growing in a mother machine channel (A) in glycerol-TrE and (B) in glucose-cas medium. The arrows in the phase images point to cells whose heatmaps are shown in Fig. 1A and in panels C and D of this figure. Time is from cell birth. The scale bar for all images is 2 μm. (C) Kymographs of fluorescent and phase signals for a representative cell grown in M9 medium supplemented with glucose-cas. Red corresponds to high and blue to low-intensity values. Black marks regions outside the cell. Dashed vertical lines indicate cell division events. The arrows indicate event timings as determined by an automated algorithm. (D) Midcell intensity traces of ZapA-mCherry (top), Ypet-FtsN (middle), and phase signal (bottom) of the cell shown on the kymograph. The intensity traces have been collected from about 0.5 μm wide band in the cell middle. The arrows show the timing for the formation of the persistent Z ring (Tz) and the time of FtsN recruitment (Tn) at midcell. a.u., arbitrary unit. Download FIG S3, EPS file, 1.3 MB. Copyright © 2022 Männik et al. 2022 Männik et al. https://creativecommons.org/licenses/by/4.0/ This content is distributed under the terms of the Creative Commons Attribution 4.0 International license.

10.1128/mbio.02017-22.8 TABLE S1 Cell characteristics data of strains used in the study (mean ± SD). Download Table S1, DOCX file, 0.01 MB. Copyright © 2022 Männik et al. 2022 Männik et al. https://creativecommons.org/licenses/by/4.0/ This content is distributed under the terms of the Creative Commons Attribution 4.0 International license.

Ypet-FtsN did not show significant colocalization with ZapA-mCherry at the midcell right after the persistent Z ring formed (marked by Tz in Fig. 1A). Only after a distinct delay did FtsN start to accumulate to a well-defined midcell band. Once started, the ensuing accumulation of FtsN was rapid compared to the accumulation of ZapA (Fig. 1A and B, middle). The initial increase in the amount of FtsN at midcell was accompanied by an increase in the midcell phase signal (Fig. 1A and B, bottom), indicating the onset of constriction approximately coincided with FtsN midcell recruitment as concluded before (16, 18, 20, 40).

**FIG 2:**
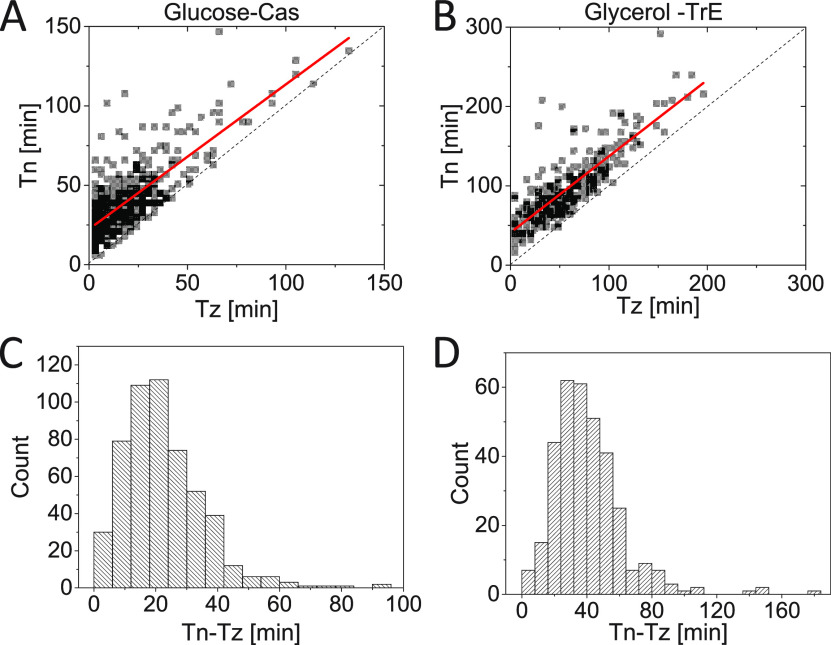
The time delay between the FtsZ/ZapA ring and N ring formation. (A and B) Timing of the formation of a persistent Z ring, *Tz*, versus timing for midcell accumulation of FtsN, *Tn*, for cells grown in M9 minimal media supplemented with (A) glucose-cas (*N* = 526) and (B) glycerol-TrE (N = 339). The solid red lines are the linear fit to the data *Tn* = (0.91 ± 0.03) *Tz* + (22 ± 0.8) *min*, (*R* = 0.77) for glucose-cas and *Tn* = 0.96 ± 0.03 *Tz* + (41 ± 2.2) *min*, (*R* = 0.84) for glycerol-TrE cells. The dashed black line corresponds to *Tn* = *Tz*. (C and D) Distributions of time delays between the Z ring and N ring formation (*Tn* − *Tz*) for cells grown in (C) glucose-cas media (21 ± 13 *min*; *mean* ± *SD*) and (D) in glycerol-TrE media (39 ± 22 min; *mean* ± *SD*).

We then determined the timing of Z ring formation (Tz) and the onset of FtsN accumulation at midcell (Tn) from time-lapse images (Fig. 1A) using an automated algorithm (Text S1). We refer to time Tn as the time when an N ring is first observed, and Tz indicates the time when a persistent Z ring forms. From *Tz* on ZapA/FtsZ protofilament assemblies are continuously present in the cell until shortly before daughter cells separate. Note that in these relatively slow frame-rate time-lapse measurements, there are some uncertainties in distinguishing persistent ZapA/FtsZ assemblies from transient ones (5). However, a fully automated and traceable approach allowed us to determine this timing consistently from measurement to measurement (Text S1). The timings of Tn and Tz were well-correlated with each other in both growth conditions (Fig. 2A and B), with a Pearson correlation R=0.77 for moderately fast and R=0.84 for slow-growth conditions. However, at least part of this correlation can be explained by the fact that FtsZ protofilament assembly is required to recruit FtsN. At the same time, there was a significant lag time from the onset of Z ring formation to the onset of FtsN recruitment (Fig. 2C and D). The average lag time (all averages will be denoted by <>) was about a quarter of the cell cycle time for both media conditions (<(Tn−Tz)/Td>=27±17% in glucose-cas; 27±15% in glycerol-TrE media) (Table S1). This long lag time shows midcell formation of a persistent Z ring alone is not sufficient for the recruitment of FtsN.

### ZapA fraction at midcell reaches a maximum at the start of the FtsN recruitment to the divisome.

**FIG 3:**
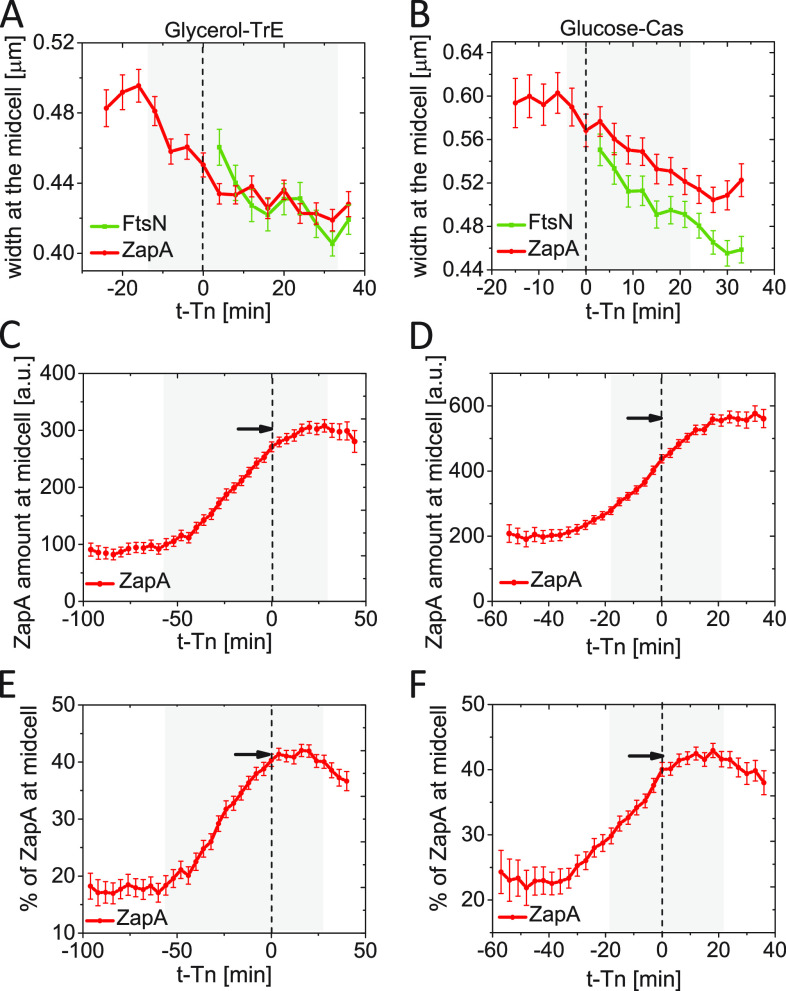
Kinetics of ZapA accumulation at about the time when FtsN is recruited at midcell. (A and B) The width of ZapA-mCherry and Ypet-FtsN accumulations at midcell as a function of time. Time zero corresponds to *Tn* (indicated by a dashed vertical line). (A) Slow-growing cells in glycerol-TrE media (*N* = 339) and (B) moderately fast-growing cells in glucose-cas media (*N* = 526). The shaded area marks the region where the number of cells analyzed is no less than 10% of its maximal value. (C and D) The excess fluorescent intensity of ZapA-mCherry at midcell as a function of time from FtsN recruitment at midcell for (C) slow-growing and (D) moderately fast-growing cells. The excess fluorescence intensity is proportional to the number of excess ZapA present at the midcell. The arrow points to the maximum level. (E and F) The excess fraction (percentage) of ZapA at midcell as a function of time from FtsN recruitment at midcell for (E) slow-growing and (F) moderately fast-growing cells. The arrow points to the maximum. All error bars represent 95% confidence intervals. The procedures to determine the width of the midcell accumulations and excess fluorescence intensity and fraction are described in Text S1.

Why is the midcell recruitment of FtsN delayed by about a quarter of the cell cycle, and what event is needed for the recruitment of FtsN to occur? It has been argued the onset of constriction in Bacillus subtilis follows the condensation of FtsZ protofilaments in the middle of the cell (48, 49). To test if a similar process also holds in E. coli, we determined the width of the Z ring and the N ring at the time of the onset of constriction (Tn). The width reflects the spatial spread of FtsZ protofilaments along the long axis of the cell. However, the reported number is larger than the actual spread because of the width of the point-spread function (PSF) of the microscope. In both growth conditions, the width of midcell ZapA-mCherry accumulations started to decrease before the onset of constriction and continued to decrease throughout the constriction period (Fig. 3A and B). The width of the N ring also decreased throughout the constriction period. Thus, there was no sharp condensation in the longitudinal distribution of ZapA/FtsZ before or at the onset of constriction, as seen in B. subtilis. However, the above analysis is not able to detect condensation at spatial scales much smaller than the width of PSF (~250 nm). Accordingly, a local condensation of FtsZ protofilaments at the onset of constriction remains a possibility.

It has also been proposed FtsZ must accumulate to some threshold number at the Z ring to initiate cell division (50). To test this idea further, we determined how the number of ZapA molecules varied at midcell as a function of time at about time Tn. In both growth conditions, the number of ZapAs in the divisome increased linearly in time before Tn but also increased 15 to 20 min past it (Fig. 3C and D). The increase in ZapA/FtsZ beyond Tn is not consistent with the idea of their threshold accumulation. Potentially, the further increase past Tn could have arisen as an artifact of fluorophore maturation if the concentration of ZapA-mCherry had varied within the cell cycle. However, we found this variation small (<10%).

We subsequently investigated how the excess fraction of ZapA at the midcell varies as a function of time in the vicinity of Tn. The excess fraction is defined as the percentage ZapA at the midcell band from which the percentage at the quarter position of the cell has been subtracted (for details, see Text S1 and Fig. S1D). This fraction corresponds to the cellular fraction of ZapA in the Z ring when the latter is present. We found the population-average excess fraction also increased about linearly after the formation of a persistent Z ring (Fig. 3E and F). At time Tn, the fraction stopped increasing in both growth conditions and plateaued at its highest value (43% in both growth conditions). So, the threshold accumulation of a relative *fraction* of ZapA holds in both growth conditions. The same behavior was also confirmed by plotting Tn versus this fraction and observing these two quantities showed almost zero correlation (Fig. S4A and B). At the same time, the correlation between Tn and the absolute amount of FtsN at the midcell at Tn was higher (Fig. S4C and D). It is unclear why the fraction of ZapA should reach a threshold value rather than the total number of ZapA molecules in the divisome at the onset of constriction. Furthermore, the coefficient of variation for both the fraction of ZapA and the total number of ZapAs in the midcell Z ring was about 0.3 at time Tn (based on data in Fig. S4), indicating if there is a threshold, then it is poorly defined in the cell population. An alternative explanation for plateauing is that at time *Tn* some change in the divisome prevents a further increase in the FtsZ amount in the Z ring. Rather than being a cause for the onset of constriction, the threshold accumulation may be a consequence of divisome maturation.

10.1128/mbio.02017-22.4 FIG S4 The relative amount of ZapA in the divisome reaches the threshold but not the total number of ZapA molecules. (A and B) Correlations between the percentage of ZapA in the Z ring at FtsN recruitment time (Tn) and Tn recruitment at midcell for cells grown in M9 minimal media supplemented with (A) glycerol-TrE (*N* = 339) and (B) glucose-cas (N = 526). The scatter plot is colored by point density. The solid red lines are the linear fit to the data *Tn* = 1.5 · 10^− 5^ *Tz* + 0.4, *R* = 0.006; *p* = 0.91) for glycerol-TrE and (*Tn* = 6.1 · 10^−4^ *Tz* + 0.37, *R* = 0.1; *p* = 0.01) for glucose-cas cells. The *P-*values indicate the probability that the slope of the linear fit is zero. (C and D) Correlations between the amount of ZapA in the Z ring at FtsN recruitment time at midcell (*Tn*) and FtsN recruitment time at midcell for cells grown in M9 minimal media supplemented with (C) glycerol-TrE and (D) glucose-cas. The scatter plot is colored by point density. The solid red lines are the linear fit to the data (*Tn* = 0.3 *Tz*+ 246, *R* = 0.14; *p* = 0.008) for glycerol-TrE and (*Tn* = 1.95 *Tz* + 355, *R* = 0.3; *p* = 10^−10^) for glucose-cas cells. Download FIG S4, EPS file, 0.7 MB. Copyright © 2022 Männik et al. 2022 Männik et al. https://creativecommons.org/licenses/by/4.0/ This content is distributed under the terms of the Creative Commons Attribution 4.0 International license.

### Constriction starts at the first distinct stage of the FtsN recruitment.

**FIG 4:**
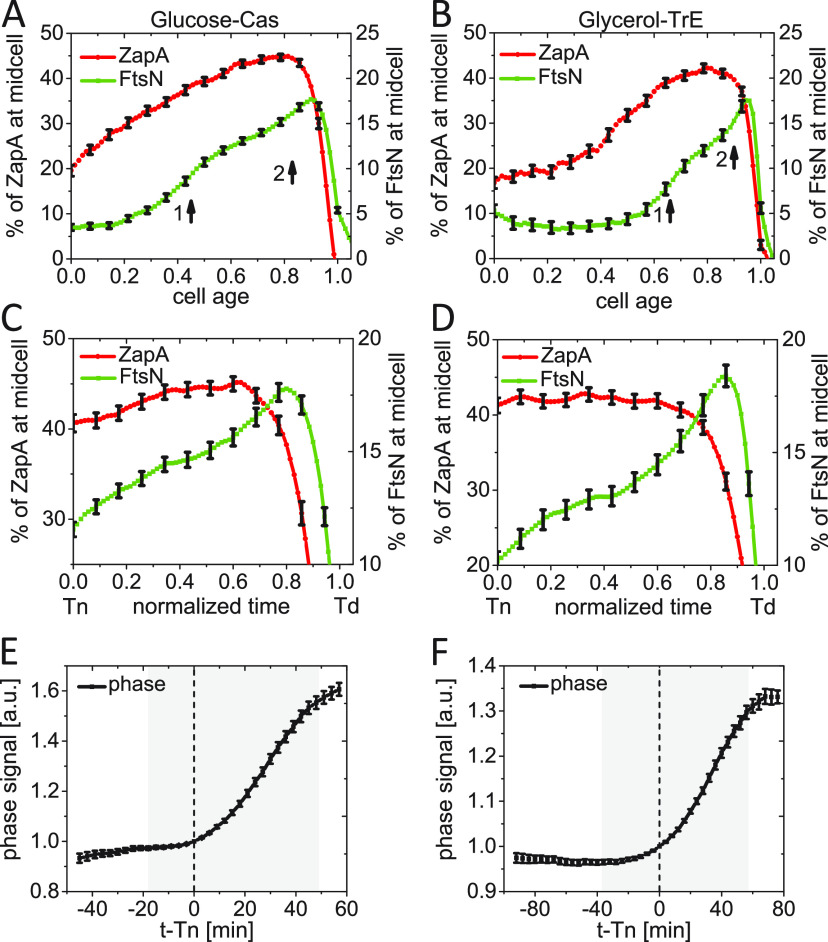
Constriction starts at the first distinct stage of FtsN recruitment. (A and B) The excess fraction of ZapA-mCherry (left axis) and Ypet-FtsN (right axis) at midcell as a function of cell age for (A) moderately fast-growing (glucose-cas media, *N* = 526), and (B) slow-growing cells (glycerol media, *N* = 339). Arrows show the approximate start of stages 1 and 2 based on local maxima in the slope of the curve (Fig. S4A and B). (C and D) The same as in (A and B) but zooming in a shorter time interval from *Tn* to *Td*. The normalized time is defined as (*Time* − *Tn*)/(*Td* − *Tn*). (E and F) Phase signal intensity at midcell as a function of time from FtsN recruitment at midcell for (E) moderately fast-growing and (F) slow-growing cells. Time zero corresponds to *Tn* (indicated by a dashed vertical line). The shaded area marks the region where the number of cells analyzed is no less than 10% of its maximal value. All error bars correspond to 95% confidence intervals (for clarity, only every fifth point is shown in panels A to D).

Our single-cell data (cf. Fig. 1B, middle) is indicative that the recruitment of FtsN occurs in two stages during the cell cycle, although single-cell signals show large fluctuations. This finding would support the idea that FtsN is first weakly recruited to the divisome via FtsA and later more strongly via its SPOR domain once septal PG synthesis starts (16, 28, 33). To investigate this idea further, we averaged the single-cell data over the cell population to remove the large fluctuations in protein numbers inherent to single-cell data. The population-averaged excess midcell accumulation of FtsN in a 0.75 μm wide band at the midcell showed a constant level at the early stages of the cell cycle (7 to 10%) and then two distinct increases when plotted as a function of cell age (Fig. 4A and B). The initial constant region corresponded to a mostly diffuse distribution of FtsN throughout the plasma membrane with a broad maximum of this distribution at the midcell (cf. Fig. 1C and D). The first rise in the FtsN midcell amount, as determined by the peak in the first derivative of the curve, occurs at about 0.45Td in glucose-cas and 0.66Td in glycerol-TrE measurements (Fig. S5A and B). The timings of the FtsN increase are close to the population-averaged times <*Tn*>, which are (0.48 ± 0.11) *Td* and (0.65 ± 0.14) *Td* (*mean* ± *SD*) in these growth conditions. Such an increase was missing in the ZapA-mCherry signal (Fig. S5C, D). Note the plateau region apparent in Fig. 3E and F for the ZapA-mCherry signal is sloping in Fig. 4A and B because of the large spread in Tn timings within the cell population. There was also a second distinct increase in FtsN accumulation in the population-averaged data. It started at about 0.83Td for cells in the glucose-cas and 0.90Td for cells in the glycerol-TrE medium (Fig. 4A and B; Fig. S5A and B). This increase was accompanied by a *decrease* in the relative and absolute amount of ZapA/FtsZ in the Z ring (Fig. 4C and D; Fig. S5E and F). An increase in the FtsN midcell numbers, while there was a decrease in the ZapA/FtsZ numbers, indicates FtsN is recruited independently of ZapA/FtsZ during this stage. Such recruitment may be due to the binding of the FtsN SPOR domain to denuded glycan strands, to FtsI and/or FtsBLQ. Interestingly, when the recruitment kinetics was plotted as a function of (absolute) time instead of cell age (relative time), then the first stage had almost the same duration in the two studied growth conditions (Fig. S5G and H). The same also applied to the second stage (Fig. S5G and H). The combined average time to constrict is thus almost the same in these two growth conditions, even though the doubling times were about 2-fold different (Table S1).

10.1128/mbio.02017-22.5 FIG S5 FtsN shows two distinct stages of recruitment. (A and B) The first derivative of the excess fraction of Ypet-FtsN at midcell versus cell age in (A) glucose-cas and (B) glycerol-TrE media, respectively (black line and markers). The curve from which the derivative has been taken is shown by green points (corresponding scale on the right). The latter curves are also shown in Fig. 4A and B. The vertical arrows show the approximate maxima for the first derivative curve. These maxima correspond to the beginnings of stages 1 and 2 of FtsN recruitment. (C and D) The first derivative of the excess fraction of ZapA-mCherry at midcell versus cell age in (A) glucose-cas and (B) glycerol-TrE media, respectively (black line and markers). The curve from which the derivative has been taken is indicated by red points (corresponding scale on the right). (E and F) The absolute excess amount of ZapA-mCherry (left axes) and Ypet-FtsN (right axes) at midcell as a function of normalized time from FtsN recruitment (*Tn*) (*Td*) for cells in (E) glucose-cas (*N* = 500) and (F) glycerol-TrE medium (*N* = 300). The normalized time is defined as (*Time−Tn*)/(*Td−Tn*). (G) Excess fraction of FtsN at midcell (0.75 μm wide band) as a function of (absolute) time for cells grown in glycerol-TrE (*N* = 339) and glucose-cas media (*N *= 526). The dashed lines show the number of cells (right axis) analyzed for every data point. For the glucose-cas condition; the shaded area marks the region where the number of cells analyzed does not vary more than 10%. For the glycerol-TrE condition, all data points show less than 10% variation. (H) Distribution of constriction duration times (*Td−Tn*) for cells grown in glycerol-TrE media (47±14; mean±SD) and glucose-cas media (40±15;mean±SD). Download FIG S5, EPS file, 0.6 MB. Copyright © 2022 Männik et al. 2022 Männik et al. https://creativecommons.org/licenses/by/4.0/ This content is distributed under the terms of the Creative Commons Attribution 4.0 International license.

Our measurements show only a relatively small fraction of FtsN is recruited to the divisome even at its peak recruitment. At the peak of the second stage, we found the number of FtsNs in the divisome was just 16% of the total number of FtsN molecules present in the cell in glycerol-TrE and 18% in glucose-cas medium (Fig. 4A to D). Similar percentages were also found by others (43). It is possible the percentage does not reflect the total partitioning ratio of FtsN between the divisome and the rest of the cell taken that truncated Ypet-FtsN proteins are present.

Next, we investigated how the two stages of FtsN recruitment are related to the onset of constriction. To that end, we followed the phase signal at midcell. The change in the latter is approximately proportional to the change in the amount of the dry biomass at the midcell (51). As the septum starts to constrict, the amount of dry biomass in the cell center decreases, leading to an increase in the phase signal in the negative phase-contrast imaging that we used (cf. Fig. 1B, bottom). The increase in the phase signal (indicating constriction) approximately coincided with the onset of the first stage of the FtsN recruitment (within about ±3 min glucose-cas and ±8 min in glycerol-TrE medium) when we aligned phase signals of single cells relative to their Tn (Fig. 4E and F). A similar behavior could also be seen when the 1st derivative of the phase signal was plotted as a function of cell age (Fig. S6A and B). Thus, these measurements indicate the onset of constriction starts at the first stage of FtsN recruitment to the divisome.

10.1128/mbio.02017-22.6 FIG S6 Midcell recruitment of FtsN compared with the first derivative of the phase signal and comparison of changes in FtsN and ZapA midcell accumulations upon FtsA overexpression with the WT strain (JM144). (A and B) The time derivative of the phase signal at the midcell (left axis) compared with the excess fraction (percentage) of FtsN at the midcell (right axis) as a function of cell age for cells in (A) glucose-cas and (B) glycerol-TrE media. The arrows are the same as in Fig. S5 panels A and B, respectively. (C and D) The excess fraction of (C) ZapA-mCherry and (D) Ypet-FtsN for the WT strain (JM144), and for strain JM150 that carries plasmid pSEB306+. In the latter case, the curves before and after induction are shown. Some effects from leaky expression of FtsA in uninduced conditions can already be seen in the ZapA-mCherry signal. E-F The width of (E) ZapA-mCherry and (F) Ypet-FtsN midcell accumulations for the WT strain (JM144), and for strain JM150 that carries plasmid pSEB306+. In the latter case, the curves before and after induction are shown. All traces were aligned to the time of FtsN recruitment at midcell, *t−Tn*=0 (shown by a dashed line). The shaded area marks the region where the number of cells analyzed does not vary more than 10%. The error bars represent 95% confidence intervals. Download FIG S6, EPS file, 0.5 MB. Copyright © 2022 Männik et al. 2022 Männik et al. https://creativecommons.org/licenses/by/4.0/ This content is distributed under the terms of the Creative Commons Attribution 4.0 International license.

### Upregulation of FtsA affects recruitment of ZapA/FtsZ and condensation of the Z ring but not the N ring.

**FIG 5:**
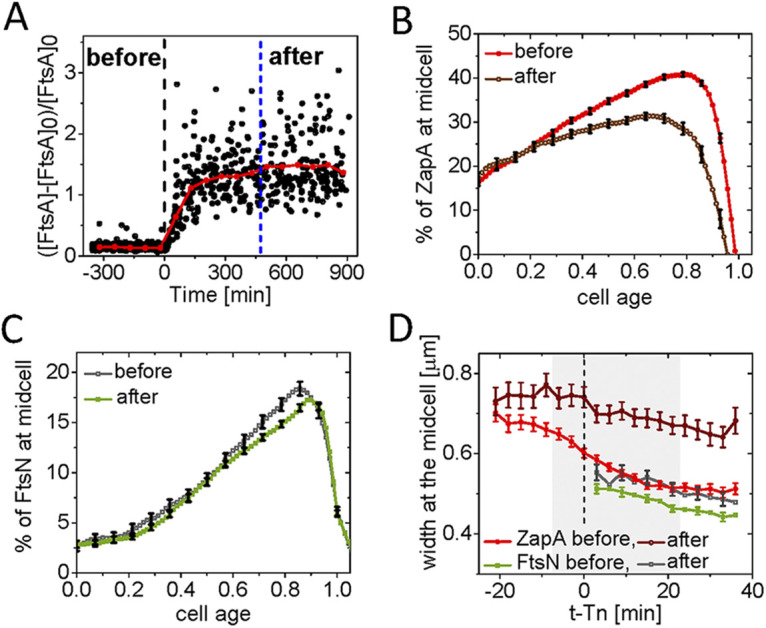
Changes in FtsN and ZapA midcell accumulations upon FtsA overexpression. (A) Relative increase in the concentration of FtsA, [FtsA], at cell birth before and after induction with 100 μM IPTG as inferred from monitoring GFP reporter expressed from pDSW210 (strain JM149). The shown increase is relative to WT FtsA concentration, [FtsA]_0_, which is determined by Western blotting. Time zero (black dashed vertical line) corresponds to the start of the induction. *N* = 1,470. (B) The excess fraction of ZapA-mCherry at midcell before and after overexpression of FtsA as a function of cell cycle time. For cells termed “after,” ZapA amounts were analyzed when the cell growth reached a new steady-state (indicated by a blue vertical dashed line in panel A). Cells were grown in M9 glucose-cas media. The error bars represent 95% confidence intervals (for clarity, only every fifth point is shown). *N* = 660 (before); *N* = 223 (after). (C) The excess fraction of Ypet-FtsN at midcell before and after overexpression of FtsA as a function of cell cycle time. Conditions as above for ZapA-mCherry in (B). (D) The width of ZapA-mCherry and Ypet-FtsN accumulations at midcell as a function of time before and after overexpression of FtsA. Midcell traces of ZapA-mCherry and Ypet-FtsN were aligned at the time of FtsN recruitment at midcell, *t* − *Tn* = 0, is marked by a dashed line. The shaded area marks the region where the number of cells analyzed does not vary more than 10%. The error bars represent 95% confidence intervals (for clarity, only every second point is shown for FtsN).

To further understand what role FtsA plays in the recruitment of FtsN and early divisome proteins, we introduced an extra copy of FtsA on a plasmid (pSEB306+) containing an isopropyl-β-d-thiogalactoside (IPTG) inducible P_trc_ promoter into our strain (52, 53). FtsA expression from this plasmid was leaky and more so in slow-growth conditions in glycerol medium which lacks catabolite repression. Therefore, we focus here only on the results from the moderately fast-growth condition in glucose-cas medium. Under our experimental conditions in glucose-cas medium, this plasmid led to upregulation of the concentration of FtsA in the cell by about 150% based on Western blotting (Fig. S2B). In addition to the above-mentioned strain, we constructed a reference strain expressing GFP instead of FtsA from pDSW210 in nonlabeled WT background cells (BW27783). We simultaneously imaged this reference strain (JM149) in the same microfluidic chip with the above-described strain of interest (JM150). The total fluorescence intensity of GFP in the reference strain allowed us to estimate the number of FtsA molecules synthesized from plasmid pSEB306+ before and after IPTG induction revealing that leakage from the plasmid accounted for less than 15% of the native protein level in uninduced conditions (Fig. 5A). The induced condition resulted in a 150% increase in the level of FtsA, which led to an increase in the cell length by about 10% (Table S1). This behavior is different from the high-level expression of FtsA that inhibits cell septation and causes the formation of filamentous cells (54–56).

The main effect observed after overexpression of FtsA was a significantly reduced amount of ZapA-mCherry in the divisome (Fig. 5B; Fig. S6C). In the first quarter of the cell cycle, the amount of ZapA-mCherry at midcell was not affected. In later parts of the cell cycle, the midcell ZapA fraction decreases from about 41% at its peak to 30%. At the same time, strikingly, the fraction of FtsN at midcell remained almost unaffected by FtsA upregulation throughout the cell cycle (Fig. 5C; Fig. S6D). Thus, FtsA upregulation by 150% does not affect recruitment of FtsN to midcell, but it has a significant effect on ZapA/FtsZ recruitment.

We also investigated how the increased FtsA affected the condensation of the Z ring. Based on *in vitro* measurements, it was suggested FtsA acts as an anti-bundling agent by forming minirings (57, 58). We, therefore, compared the changes in the width of both the Z and N rings before and after the onset of constriction (Tn). Before FtsA induction, both the Z and N rings condensed to a similar width as in the WT strain without the plasmid (Fig. 5D; Fig. S6E and F). However, in the FtsA upregulated condition, the distribution of FtsZ protofilaments along the long axes of the cell remained wide and did not condense as also observed before (59). In contrast, the N ring was not affected and appeared similar to WT cells (Fig. 5D). This finding indicates FtsN and ZapA/FtsZ are present in spatially separated complexes. These conclusions agree with previous observations that the early Z ring proteins were separated from proteins in the PG synthesizing machinery along the radial and circumferential directions (19, 44, 60). Here, our data show separation can also occur along the cell's long axis, at least in FtsA upregulated conditions. Furthermore, the finding that the ZapA-mCherry (FtsZ)-ring stays broad and does not condense suggests FtsA upregulation antagonizes FtsZ protofilament bundling. It may also be interesting to point out the failure of the ZapA signal to condense properly before/during constriction (Fig. 5D) seems to have little effect on the timing of constriction initiation (Tn) or on the constriction period itself (Td−Tn), under these conditions (Table S1) further supporting the idea FtsZ protofilament condensation is not obligatory to trigger the onset of constriction.

### FtsA* leads to earlier recruitment of FtsN but not to earlier constriction.

It has been proposed that FtsA self-interaction prevents the recruitment of downstream proteins, including FtsN, to the divisome (29, 30, 61). The FtsA mutant R286W (FtsA*) has a reduced ability to oligomerize but still interacts normally with FtsZ (38, 61, 62). To further understand how disruption of the FtsA self-interaction affects FtsN recruitment kinetics, we upregulated FtsA* in the WT FtsA background. To that end, we replaced *ftsA* with ftsA* in plasmid pDSW210 (pSEB306+*) and repeated the experiments described in the previous section.

**FIG 6:**
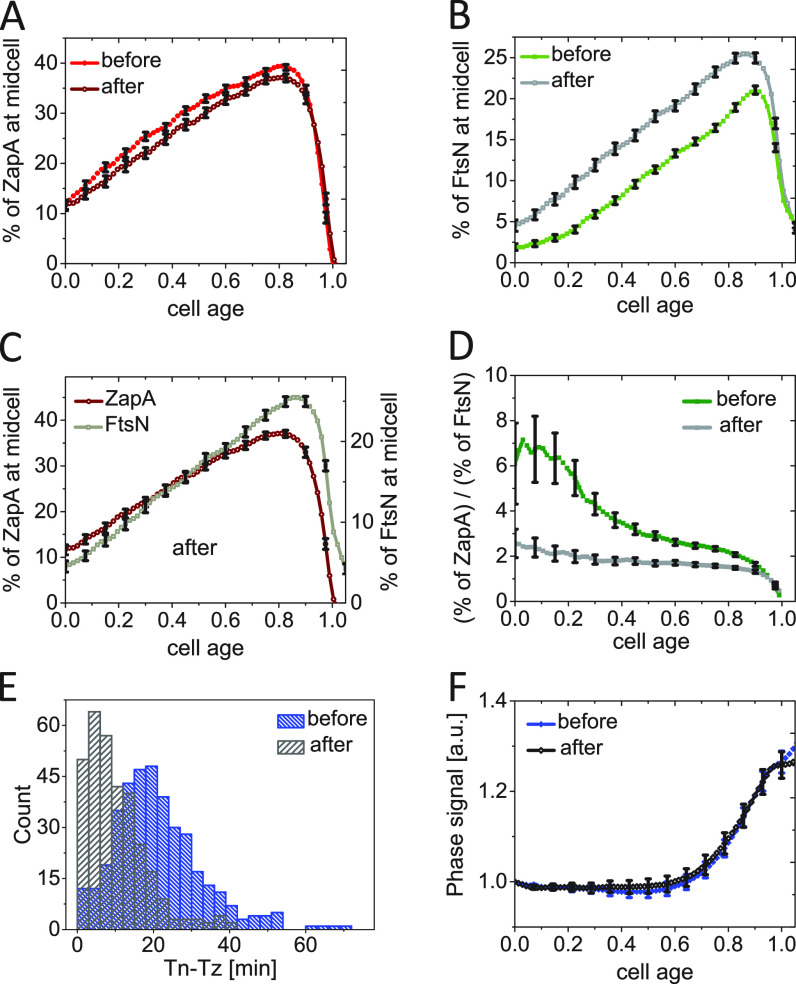
Changes in FtsN and ZapA midcell accumulations upon FtsA* (FtsA^R286W^) overexpression. (A) The excess fraction of ZapA-mCherry at midcell before and after overexpression of FtsA* (R286W) as a function of cell cycle time. FtsA* was expressed as an extra copy from a plasmid pSEB306+* by the addition of 100 μM IPTG in M9 glucose-cas media. For cells termed “after,” ZapA amounts were analyzed when the cell growth reached a new steady-state. All error bars represent 95% confidence intervals (for clarity, only every fifth point is shown). *N* = 381 (before); *N* = 321 (after) (B) The excess fraction of Ypet-FtsN at midcell before and after overexpression of FtsA* as a function of cell cycle time. Conditions as above for ZapA-mCherry in (A). (C) The excess fraction of ZapA-mCherry and Ypet-FtsN at midcell after upregulation of FtsA*. Both curves show approximately the same functional dependence on the cell age. (D) The ratio of ZapA-mCherry to Ypet-FtsN midcell fraction as a function of cell age before and after upregulation of FtsA*. (E) The distributions of time delay between the Z ring and N ring formation (*Tn* − *Tz*) in cells before (20 ±12 min; *mean* ± *SD*) and after (9 ± 8 min; *mean* ± *SD*) upregulation of FtsA*. (F) Phase signal intensity at midcell as a function of cell age before and after upregulation of FtsA*.

We found upregulation of FtsA* by about 150% with respect to the native FtsA levels did not affect cell doubling times but led to about a 25% shortening in cell length compared with WT (Table S1), consistent with previous observations (62). Overexpression of FtsA* slightly decreased the midcell fraction of ZapA-mCherry throughout the cell cycle (Fig. 6A; Fig. S7A). Compared with FtsA overexpression, the decrease late in the cell cycle was significantly less. Furthermore, we found the width of the midcell Z ring was not affected by overexpression of FtsA* (Fig. S7C). At the same time, significantly more Ypet-FtsN was recruited at midcell following the overexpression of FtsA*(Fig. 6B; Fig. S7B). The fraction of FtsN at midcell was almost uniformly increased throughout the cell cycle. Furthermore, it was noticeable that the midcell fraction of FtsN in the FtsA* upregulated conditions increased in the same way as the fraction of ZapA. Both fractions increased linearly from cell birth to about cell age 0.8 (Fig. 6C). The ratio of the two fractions was about a constant during this period in FtsA* upregulated conditions but varied significantly when FtsA* was not induced (Fig. 6D). Unlike FtsA, FtsA* is thus capable of recruiting FtsN to the divisome as soon as the Z ring is assembled and in proportion to the fraction of ZapA/FtsZ in the Z ring.

10.1128/mbio.02017-22.7 FIG S7 Effects of FtsA* (FtsA^R286W^) upregulation on FtsN and ZapA midcell accumulations and the width of Z and N rings. (A and B) The excess fraction of (A) ZapA-mCherry and (B) Ypet-FtsN for the WT strain (JM144), and for strain JM151 that carries plasmid pSEB306+* expressing FtsA*. In the latter case, the curves before and after induction are shown. (C) Width of Z ring/ZapA and N ring/FtsN signal at midcell in the longitudinal direction of the cell before and after overexpression of FtsA*. Midcell traces of ZapA-mCherry and Ypet-FtsN were aligned at the time of FtsN recruitment at midcell, *t−Tn*=0, marked by a dashed line. The shaded area marks the region where the number of cells analyzed varies by about 10%. The error bars represent 95% confidence intervals (for clarity, only every second point is shown); *N* = 381 (before); *N* = 321 (after). (D) The first derivative of the phase density at the midcell as a function of cell age before and after upregulation of FtsA*. The arrow shows the approximate start of stage 1 in cells before induction. The start time for stage 1 is <*Tn*> which is based on data in Table S1. Download FIG S7, EPS file, 0.5 MB. Copyright © 2022 Männik et al. 2022 Männik et al. https://creativecommons.org/licenses/by/4.0/ This content is distributed under the terms of the Creative Commons Attribution 4.0 International license.

In addition to higher midcell abundance, the timing of FtsN recruitment also shifted earlier (from 34±18 min to 26±17 min; *t* test: p = 4.1 × 10^−10^), and the delay between recruitment of ZapA and FtsN to midcell shortened from 20±12 min without FtsA* to 9 ± 8 min in its presence (Fig. 6E). The distribution of these delay times changed from one with a distinct lag time to an exponential, indicating the presence of FtsA* significantly shortens the lag. However, the earlier and more abundant recruitment of FtsN to the divisome did not lead to an earlier onset of constriction under these conditions (Fig. 6F; Fig. S7D). This result indicated that the recruitment of FtsN to the divisome is not a rate-limiting process for the onset of constriction under these conditions but reveals that FtsA* significantly advances FtsN recruitment throughout the cell cycle.

## DISCUSSION

**FIG 7:**
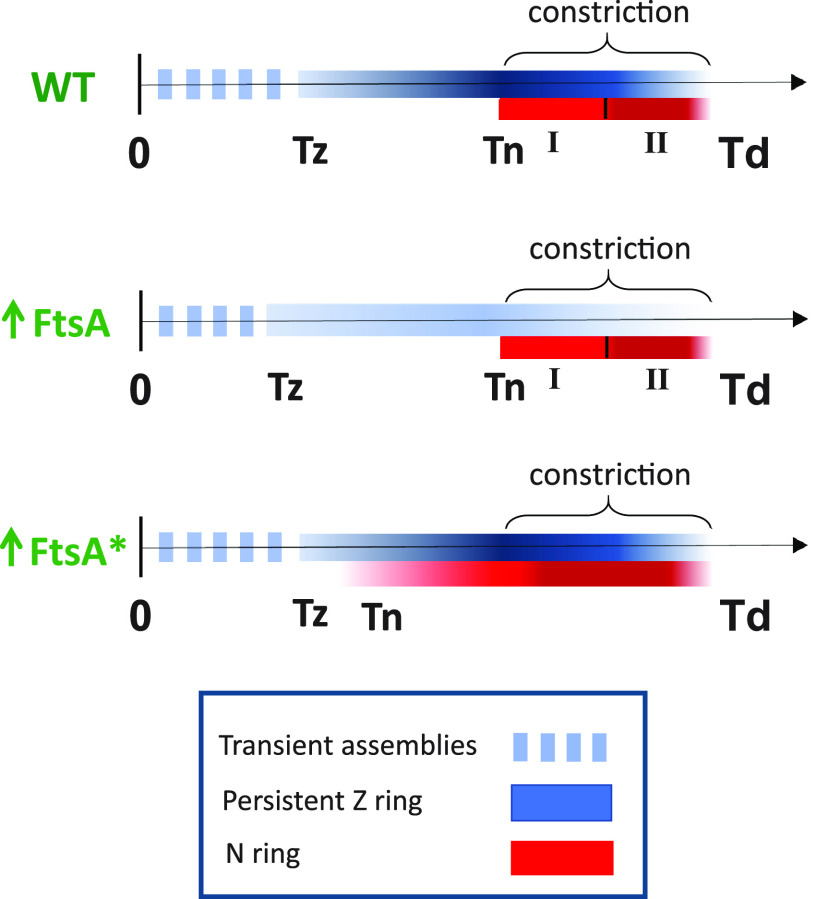
Summary of cell cycle dependent recruitment of ZapA-mCherry (blue) and Ypet-FtsN (red) to midcell in WT cells, and in FtsA and FtsA* upregulated conditions. The intensity of the colors reflects qualitatively the fractions of ZapA-mCherry and Ypet-FtsN at the midcell. I and II indicate the two stages of FtsN recruitment.

FtsN has been implicated as a trigger for the onset of constriction (16, 18, 20). Here, we studied its recruitment kinetics to the divisome using an early cell division protein ZapA as a reference after verifying the latter is a good proxy for FtsZ. Our data show, unlike the gradual increase of ZapA/FtsZ at midcell over about a quarter of the cell cycle, the recruitment of FtsN occurs abruptly over much shorter time scales and, on average, about a quarter of the cell cycle later than when the persistent Z ring forms (summarized in Fig. 7). The question then arises as to what is responsible for the delay in FtsN recruitment following the formation of the Z ring? A long delay between the formation of a persistent Z ring (Tz) at midcell and an abrupt accumulation of FtsN (Tn) suggests there is some cell cycle checkpoint between the formation of a persistent Z ring and the onset of constriction.

### Is the onset of constriction triggered by the change in FtsZ protofilament assembly at the midcell?

What could be responsible for the delay between Tz and Tn? The putative mechanisms proposed for a checkpoint for Tn so far include the change in the polymerization state of FtsZ (50), its higher-order assembly (48, 49), or that of FtsA (29, 30, 58), or simply recruitment of FtsN itself (17). To further investigate the idea that FtsZ dynamics drive the checkpoint, we followed the amount of ZapA/FtsZ and the effective width of the septal ring before and after the onset of constriction. Our data show condensation of FtsZ protofilament assemblies started before the onset of constriction and continued well past it. Unlike what was observed in B. subtilis cells (49, 63), we did not observe a change in FtsZ protofilament condensation at the onset of constriction. It is possible the mechanism triggering the onset of constriction is different in these two organisms, although many of the essential divisome components are homologous to each other. An exception is FtsN which does not have a homolog in B. subtilis (64).

We also tested the hypothesis that the number of FtsZ molecules needs to reach some threshold value at midcell to initiate constriction (50). Our data show ZapA/FtsZ numbers at the midcell approach a plateau only 15 to 20 min after Tn in both growth conditions (Fig. 3C and D). Furthermore, at this time in the cell cycle the amount of ZapA/FtsZ at midcell correlates with the timing of N ring formation (Fig. S4C and D). This latter finding contradicts the threshold accumulation model for ZapA/FtsZ. At the same time, the fraction of ZapA/FtsZ in the Z ring (about 42%) approached a plateau at *Tn* in both growth conditions (Fig. 3E and F), and the fraction of ZapA/FtsZ was much less well correlated with Tn timing (Fig. S4A and B). It is unclear how the fraction of ZapA/FtsZ in the Z ring could be a signal for the recruitment of FtsN and the onset of constriction. A more likely explanation is the constant fraction of ZapA/FtsZ at midcell indicates the divisome has matured to a functional form for PG synthesis. ZapA/FtsZ in this mature divisome appears to be in a quasi-equilibrium with the remaining population of ZapA/FtsZ in the cytosol and on other regions of the inner membrane. Quantitatively this equilibrium is determined by an equilibrium constant. As the cell synthesizes more ZapA/FtsZ, it is incorporated into a divisome in proportion to this equilibrium constant. The constant fraction thus may be the consequence of divisome maturation by some unrelated process rather than the cause, but further work is warranted to support this claim.

### Recruitment of FtsN is not rate-limiting for the onset of constriction in FtsA* background.

We found FtsN was recruited earlier in FtsA* overexpression conditions (Fig. 7). The early recruitment of FtsN in this strain can be explained by an increased number of FtsA* molecules with a free 1C domain to interact with FtsN because FtsA* is defective in self-interaction, which would mask the 1C domain (38, 61, 62). Although FtsN is recruited early in the cell cycle under these conditions, the onset of constriction still occurs at the same time in the cell cycle as in WT cells. The presence of FtsN in the septum is thus alone not sufficient to trigger the onset of constriction. This finding suggests the concentration of FtsN is not a rate-limiting for the onset of constriction in this strain and instead, some other process holds back the maturation of the divisome in the FtsA* background. The same conclusion may also hold for WT cells. It is possible that instead of FtsN the recruitment of FtsWI or possibly some other maturation process is rate-limiting for the onset of constriction.

### Decoupling of FtsN midcell accumulations from FtsZ protofilaments.

Our data (Fig. 5) show after FtsA overexpression, the fraction of ZapA/FtsZ in the Z ring decreased, and the ring did not condense at the onset of constriction as it did in WT cells. This mild effect of increased FtsA on Z ring morphology is consistent with the disassembly of Z rings that occurs when FtsA is increased 10-fold (54, 59, 65). What is surprising is that the N rings assembled normally. This finding can be explained if FtsN in the septal ring is not physically linked to FtsZ protofilaments. Evidence for decoupling FtsN and FtsZ protofilaments has also been documented in other measurements where their localization in the radial direction was examined (44) and from recent single-molecule studies (43). FtsN appears thus to be part of an active divisome complex involved in septal PG synthesis that is not physically linked to FtsZ protofilaments.

Our data on the upregulation of FtsA and FtsA* could imply FtsA forms different higher-order structures when initially linked to FtsZ protofilaments and when part of an active divisome complex. If FtsA would form the same polymeric structures in both complexes, then these structures should respond the same way to its upregulation. However, this was not the case in the experiments on FtsA upregulation which led to the downregulation of ZapA but did not change the amount of FtsN at the midcell. *In vitro* FtsA forms 12-member minirings (58) and antiparallel actin-like filaments on membranes (66, 67). These different FtsA structures may reflect different roles of FtsA in the assembly and activation of the divisome (67).

### Two different stages in the recruitment of FtsN to midcell.

Our data reveal two distinct stages in FtsN recruitment to midcell, both involving septal PG synthesis (Fig. 7). We observed the first FtsN recruitment/accumulation occurs approximately at the onset of the constriction (Fig. 4C and D; Fig. S5). This finding may imply the arrival of FtsN results in rapid activation of septal PG synthesis, although based on the time-resolution of our measurements, it is also possible initial septal PG synthesis starts first and FtsN is recruited thereafter. In the former scenario, FtsN could be recruited first via its binding to FtsA (27, 28, 30). However, in this case, there is no extended period when FtsN lingers in the divisome just due to its sole binding to FtsA because once septal PG synthesis starts, FtsN can bind to denuded peptidoglycan strands also via its SPOR domain. This is presumably stronger binding than the binding of FtsN to FtsA, but the former is not negligible because cells without the FtsN cytoplasmic domain or the FtsN D5N mutants show a division defect and elongated phenotype (29, 30).

The second distinctive stage of FtsN accumulation occurs late in the cell cycle, about 15 min before the separation of daughter cells in both growth conditions. There seems to be some additional mechanism involved in this stage, in addition to the SPOR-domain-related self-enhanced recruitment of FtsN. Interestingly, the start of the second stage of FtsN recruitment occurs approximately when the amount of ZapA/FtsZ in the septal ring starts to decrease. While this could be a mere coincidence, it appears FtsZ protofilament-involved complexes may sequester away some of the FtsN binding partners such as FtsQLB from the divisome complexes. Once FtsZ protofilaments at the midcell become unstable, perhaps because of high membrane curvature at the site of constriction, fewer FtsQLB complexes are associated with FtsZ protofilaments, and more can be directly recruited by the divisome complexes.

Septal cell wall synthesis proceeds at the end of the division process without any FtsZ protofilaments present at the division site (Fig. 4A to D), as also observed earlier (60). It is likely FtsN and, to a lesser degree DedD, which also features a SPOR domain (68), completely take over the role of positioning the divisome apparatus from FtsZ protofilaments. This positioning may be possible thanks to curvature-sensing of FtsA antiparallel filaments, which allows them to localize to regions of negative Gaussian curvature (67).

The two phases of FtsN accumulation may also correspond to the septation stages recently observed in TEM images of dividing E. coli (69). The images showed a V-shaped invagination in the early stages of septation, referred to as the constriction phase. This invagination developed an inward protrusion similar to the septum of B. subtilis in the second stage, referred to as the septation phase. Although the authors did not determine the timings of these phases in the cell cycle, their approximate durations appear similar to stages one and two observed here for FtsN recruitment.

In conclusion, our data show an abrupt accumulation of FtsN to the divisome, which occurs about 25% of cell cycle time after a persistent Z ring forms. At this time of the cell cycle, the fraction of ZapA/FtsZ in the Z ring approaches its highest value. The fraction of FtsN at midcell shows a further distinct increase at the time when ZapA/FtsZ starts to dissociate from the septum. Furthermore, our data support the idea (43, 44) FtsN is not part of the complexes that include FtsZ protofilaments but resides in separate divisome complexes involved in septal PG synthesis. Finally, our data show the recruitment of FtsN to midcell is not rate-limiting for the onset of constriction in the FtsA* background.

## MATERIALS AND METHODS

### Media, bacterial strains, and plasmids.

Cells were grown with M9 minimal media (Teknova Inc., CA) supplemented with 0.5% glucose (Millipore Sigma, MO) and 0.2% Casamino Acids (cas, ACROS Organics) or 0.3% glycerol (Thermo Fisher Scientific) and trace metals mixture (TrE, Teknova Inc., CA) at 28°C. Unless indicated otherwise, antibiotic concentrations were as follows: ampicillin (Amp), 100 μg/mL; chloramphenicol (Cm), 25 μg/mL; kanamycin (Kan), (25 μg/mL). For induction, 100 μM IPTG was used. All E. coli strains used in the reported experiments are derivatives of K12 BW27783 obtained from the Yale Coli Genetic Stock Center (CGSC#: 12119). The strain with fluorescent fusions of ZapA-mCherry and Ypet-FtsN (JM144) was made by P1 transduction (70). All strains and plasmids used in this study are listed in Tables S2A and S2B, respectively.

10.1128/mbio.02017-22.9 TABLE S2 Strains used in this study. Download Table S2, DOCX file, 0.01 MB. Copyright © 2022 Männik et al. 2022 Männik et al. https://creativecommons.org/licenses/by/4.0/ This content is distributed under the terms of the Creative Commons Attribution 4.0 International license.

Further details for cell preparation and culture in microfluidic devices, fluorescence microscopy, image analysis, and Western blot analysis can be found in Text S1.
