# Supplementary material for: Cell Cycle-Dependent Recruitment of FtsN to the Divisome in Escherichia coli
Source: mBio. 2022 Aug 15;13(4):e02017-22. doi: 10.1128/mbio.02017-22 (PMC9426451; doi:10.1128/mbio.02017-22)
Supplement: Text S1 [file mbio.02017-22-s0001.docx]

**SI Text**

**Extended Materials and Methods**

**Cell preparation and culture in microfluidic devices**

All bacterial strains were streaked on agar plates containing M9 minimal salts supplemented with 2 mM magnesium sulfate, corresponding carbon sources, and appropriate selective antibiotics. A day before an experiment, a less than ten days old colony was inoculated into 3 ml of M9 minimal salts media supplemented with corresponding carbon sources, trace metals mixture, and casamino acids. 100 µg/ml of Amp was added to grow strains JM149, JM150 and JM151. For microscopy experiments, cells were grown to an OD600 of ~0.1 in a liquid medium and then concentrated ~100x by centrifugation in the presence of 0.075 mg/ml of BSA (Bovine Serum Albumin; Millipore Sigma, MO) to minimize clumping of the cells. For FtsA/FtsA* induction experiments, cells of reporter strain (JM149) expressing GFP from pDSW210 were prepared the same way and mixed with the strain of interest before loading to the device. The resulting solution was used to inoculate microfluidic mother machine devices. The latter was made of PDMS (polydimethylsiloxane) following a previously described procedure (71). For inoculation 2-3 µl of resuspended concentrated culture was pipetted into the main flow channel of the device. The cells were then let to populate the dead-end channels. Once these channels were sufficiently populated (about 1 hr), tubing was connected to the device, and the flow of fresh M9 medium with corresponding carbon sources, supplements, BSA (0.075 mg/ml) and Amp (100 µg/ml) when needed, was started. The flow was maintained by a NE-1000 Syringe Pump (New Era Pump Systems, NY) at 5 µl/min during the entire experiment. The cells were left to grow in channels for at least 14 hr to ensure steady-state growth. Before imaging, the second NE-1000 Syringe Pump containing 100 µM IPTG additional to M9 medium with corresponding carbon sources, supplements, BSA, and Amp was connected to microfluidic chips via a separate tubing. Before applying IPTG to induce FtsA or FtsA* expression from the plasmid, cells were imaged in regular media. After 6-8 hours of imaging, the first pump with the regular media was turned off and the second syringe pump containing regular media with IPTG was turned on without interrupting imaging. Altogether cells were imaged for 20-24 hrs.

**Fluorescence microscopy**

A Nikon Ti-E inverted fluorescence microscope (Nikon Instruments, Japan) with a 100X NA 1.40 oil immersion phase-contrast objective (Nikon Instruments, Japan) was used for imaging the bacteria. Images were captured on an iXon DU897 EMCCD camera (Andor Technology, Ireland) and recorded using NIS-Elements software (Nikon Instruments, Japan). Fluorophores were excited by a 200W Hg lamp through ND4 and ND8 neutral density filters. Chroma 41004 and 41001 filter cubes (Chroma Technology Corp., VT) were used to record mCherry and Ypet images, respectively. A motorized stage (Prior Scientific Inc., MA) and a Nikon Perfect Focus ® system were utilized throughout time-lapse imaging. Images in M9 glycerol-TrE media were obtained at 4 min frame rate and M9 glucose-cas at 3 min frame rate. The typical exposure times were 250 ms at EM gain 200 for ZapA-mCherry, 350 ms at EM gain 200 for Ypet-FtsN, and 400 ms without EM gain for the phase images.

**Image analysis**

MATLAB, along with the Image Analysis Toolbox and DipImage Toolbox (http://www.diplib.org/) were used for image analysis. In all analyses of time-lapse recordings, corrections to subpixel shifts between different frames were applied first. These shifts were determined by correlating phase-contrast images in adjacent frames. The cells were then segmented based on phase-contrast images using a custom MATLAB script. Timings of cell divisions were corrected based on the dissociation of the Ypet-FtsN label from the septum. The segmented images were then used to extract intensity line profiles along the long axes of the cell. To find an intensity line profile, 11 individual line profiles from about a 1.2 µm wide band centered on the long symmetry axes were averaged (SI Fig. S1C). Although this band is wider than the cell, some fluorescence intensity from the cell spreads beyond this band due to diffraction. However, we verified that a further increase in the width of the band did not qualitatively change the shape of any curves in any of the figures presented here. The average profile fluorescence intensity of an equally wide band, but shifted 5 µm from the cell, was subtracted (SI Fig. S1C). These background-corrected intensity line profiles were used in the following data analysis (as detailed below) and to compile heatmaps of fluorescent images, as shown in Fig. 1A and SI Fig. S3D. The background was not subtracted from the average line profiles for the phase images.

**Compilation of midcell intensity vs. time traces**

The midcell intensities for these traces were collected from about a 0.75 µm wide band (7 pixels wide; Fig. 3, 4, 5) or for shorther cells from a a 0.5 µm wide band (5 pixels wide; Fig. 1, 6) centered about the mid cell (SI Fig. S1D). From the integrated intensities in this band, the cell background intensity was subtracted except for measurements derived from the phase signals. The background intensity for subtraction was also integrated from a 0.75 µm wide band (7 pixels wide). The latter band was centered at the ¼ position from the old pole of the cell. The exact locations for the midcell band were based on the position of the Z ring during the cell cycle. This position was found from fitting a Gaussian to intensity line profiles of fluorescent labels as described in the next section. The relative positions of Gaussians from cell poles were approximately 0.5 for almost all the cells in this study.

For the plots showing “% at midcell”, the background-subtracted midcell intensity was divided by the total integrated fluorescent intensity from that cell at that time point (SI Fig. S1D). For the plots where time dependence was represented as a function of cell age, the measured times were normalized by doubling times, and a signal was interpolated at equidistant intervals for each cell using Matlab interp1 function with the linear method. The traces from individual cells were then averaged. For plots where the absolute time was plotted relative to $Tn$, the intensity traces were averaged without any interpolation. Grey areas in the latter plot mark regions where the contributing cell counts are more than 90% of their maximum value. The latter corresponds to N reported on the plot. Beyond this region, the data is biased by the higher contribution of slow-growing cells in the population.

**Determination of** $\boldsymbol{Tn}$ **and** $\boldsymbol{Tz}$ **timings and width of accumulations**

The timings were determined using fully automated analysis. First, the intensity line profiles from each frame were fitted to a Gaussian defined by

$$I(x)=a_{0}+a_{1}exp(-{4ln(2)(x-x_{0})}^{2}/{FWHM}^{2})$$

Here, $a_{0}$ is the constant background, $a_{1}$the amplitude and $x_{0}$ the centroid of the Gaussian. FWHM refers to the Full Width at Half Maximum of the Gaussian. If the fitted peak contained more than 5% (10%) of the total fluorescence signal from the cell, the FWHM of the Gaussian was between about 200 and 1000 nm, and the peak was not more than 10% off from the cell center in terms of cell length then this fitting qualified as an FtsN accumulation (or a ZapA accumulation). 5% and 10% threshold values were based on noise levels in Ypet-FtsN and ZapA-mCherry signals, respectively. These accumulations were further filtered to extract a single cluster of accumulations in each cell cycle. In this filtering, the presence of accumulations was tracked from the division frame to the birth frame. If an accumulation was missing in one frame but present in at least two previous frames, then the lack of this accumulation was ignored. Otherwise, tracking of the accumulations stopped, and the timing of the earliest accumulation present in the tracked set of points was assigned to $Tn$, ($Tz$). The width of midcell accumulations for ZapA-mCherry and Ypet-FtsN were determined from these fittings. The width of the point spread function of the microscope of about 0.32 μm was not subtracted from these curves.

**Compilation of cell age plots in Fig. 1**

The plot combines all line intensity profiles from all analyzed cells. For the compilation of this plot, the age, $a$, of a given cell in a given frame of measurement is calculated first. Then the average cell length for that age is calculated using$L\left( a \right)=<L_{birth}>2^{a}$. Here $<L_{birth}>$ is the average cell length at birth for the cell population. $<L_{birth}>= <L_{div}>/2$ where $<L_{div}>$ can be found in Table S1. While $L\left( a \right)=<L_{birth}>2^{a}$ is not exactly an accurate description of cell age-dependent growth (72) we neglect here this discrepancy for the lack of a more accurate analytic model. The intensity profile is then added to the corresponding age bin after mapping the cell length to the average cell length at this age bin using linear interpolation. Finally, the data in each age bin is divided by the number of measurements in this bin.

**Western blot analysis**

For Western blot of FtsA, overnight cultures of JM149 and JM150 were diluted to OD600 of 0.005 in fresh M9 glucose-cas medium in the presence of Amp (100 µg/ml) and grown at 28°C for about 4-6hrs to OD600 of 0.04-0.05. The cultures were diluted back to OD600 of 0.005 in M9 glucose-cas with Amp (100 µg/ml) and split into two sets of triplicates. To one set, 100 µM of IPTG (Sigma) was added. Cells grown in the glucose-cas medium were collected after 7 hours of induction (at OD600 of 0.36). The 7-hour time period was based on the procedure used in the microfluidic experiments where cells during this time reached a stable expression of FtsA from the plasmid. The collected cells were pelleted and resuspended in lysis buffer (50 mM Tris pH8, 300 mM NaCl, 10mM MgCl_2_, 1 mM EDTA) and stored at −80 °C. For lysis, cells were thawed and incubated with lysozyme (10 mg/ ml, MP Biomedicals) and DNase (1 mg/ml, ThermoFisher Scientific) in the presence of Pierce Protease Inhibitor mix (ThermoFisher Scientific). The resulting extract was mixed with SDS sample buffer (LI-COR Biosciences), boiled for 10 min, and separated by SDS-PAGE in a Mini PROTEAN Tetra Cell (Bio-Rad) using 10% polyacrylamide gels. Proteins were transferred to a low-fluorescence PVDF membrane (ThermoFisher Scientific) using a Bio-Rad Mini Trans-Blot Cell. The membrane was blocked with Odyssey® blocking buffer (LI-COR Biosciences) according to the manufacturer's protocol. Primary antibodies (anti-FtsA serum at final concentration 1:750, UK54, Lutkenhaus lab) were diluted in blocking buffer and incubated overnight at 4°C. The secondary donkey anti-rabbit IRDye 680RD antibodies were used at the dilution of 1:15,000. Membranes were incubated in secondary antibody for 45 min at RT, followed by three washes (TBST, 10 min each). Images of the near-infrared fluorescent signal were acquired using an Odyssey scanner (LI-COR Biosciences), and the bands were quantified using ImageJ software.

To compare the level of native FtsN and Ypet-FtsN, BW27783 and JM144 strains were cultured in M9 glucose-cas medium as described above for the FtsA Western blot cultures. Cells were collected, resuspended in SDS sample buffer, and boiled for 10 min before they were loaded on the SDS-PAGE gel for analysis. Western blotting and detection of Ypet-FtsN were performed as described above except for the primary antibodies. For FtsN detection, 1:350 dilution of anti-FtsN antisera (UK43; Lutkenhaus lab) in Odyssey blocking buffer was used.

Note that we also did a Western blot with anti-GFP polyclonal antibody (Invitrogen, A-11122). However, these antibodies did not bind to any of the Ypet-FtsN bands.
