# Supplementary material for: Cell Cycle-Dependent Recruitment of FtsN to the Divisome in Escherichia coli
Source: mBio. 2022 Aug 15;13(4):e02017-22. doi: 10.1128/mbio.02017-22 (PMC9426451; doi:10.1128/mbio.02017-22)
Supplement: TABLE S1 [file mbio.02017-22-st001.docx]

**Table S1:** Cell characteristics data of strains used in the study (mean ± SD).

^1^$Td$, doubling time;

^2^$Tz$, timing for the Z ring formation;

^3^$Tn,$ the onset of FtsN accumulation at midcell;

^4^ $Tn-Tz,$ delay time between FtsN accumulation at midcell and formation of the Z ring;

^5^ $Td-Tn,$ time between cell division and FtsN accumulation at midcell;

^6^ $Td-Tz,$ time between cell division and formation of the Z ring;

^7^ $L_{div}$, cell length at division

| **Strain** | **Media** | **No. of cells** | **Td^1^**  **[min]** | **Tz^2^**  **[min]** | **Tn^3^**  **[min]** | **Tn-Tz^4^**  **[min]** | **Td-Tn^5^**  **[min]** | **Td-Tz^6^**  **[min]** | **L_div_^7^**  **[µm]** |
| --- | --- | --- | --- | --- | --- | --- | --- | --- | --- |
| **WT**  **(JM144)** | glycerol-TrE | 339 | 143±45 | 57±35 | 96±41 | 39±22 | 47±14 | 86±26 | 3.33±0.30 |
| **WT**  **(JM144)** | glucose-cas | 526 | 78±28 | 17±17 | 38±20 | 21±13 | 40±15 | 61±20 | 4.00±0.53 |
| **FtsA (JM150)**  before induction | glucose-cas | 660 | 72±27 | 14±16 | 36±20 | 22±13 | 36±16 | 58±21 | 4.13±0.61 |
| **FtsA (JM150)**  after induction | glucose-cas | 223 | 77±22 | 16±20 | 37±19 | 21±18 | 40±16 | 62±22 | 4.60±0.83 |
| **FtsA* (JM151)**  before induction | glucose-cas | 381 | 78±28 | 15±14 | 34±18 | 20±12 | 43±17 | 63±22 | 3.61±0.57 |
| **FtsA* (JM151)**  after induction | glucose-cas | 321 | 83±26 | 17±16 | 26±17 | 9±8 | 57±18 | 66±18 | 3.02±0.45 |
| **BW27783**  **no labels** | glucose-cas | 318 | 76±28 | NA | NA | NA | NA | NA | 3.90±0.6 |
| **BW27783**  **no labels** | glycerol-TrE | 175 | 146±41 | NA | NA | NA | NA | NA | 3.2±0.25 |
