# Supplementary material for: Cell Cycle-Dependent Recruitment of FtsN to the Divisome in Escherichia coli
Source: mBio. 2022 Aug 15;13(4):e02017-22. doi: 10.1128/mbio.02017-22 (PMC9426451; doi:10.1128/mbio.02017-22)
Supplement: TABLE S2 [file mbio.02017-22-st002.docx]

**Table S2A.**  Strains used in this study.

| **Strain** | **Relevant genotype** | **Source** | **Construction** |
| --- | --- | --- | --- |
| BW27783 | *Δ(araD-araB)567, ΔlacZ4787*(::rrnB-3), *Δ(araH-araF)570(::frt), ΔaraEp-532::frt, hsdR514, φP_cp8_araE535, rph-1, Δ(rhaD-rhaB)568, λ^-,^* | Yale Coli Genetic Stock Center (CGSC#: 12119) |  |
| STK9 | BW27783, *ΔftsN::frt-Ypet-ftsN* | Tiruvadi-Krishnan *et al*. 2021 |  |
| TU211 | MG1655*, lacIZYA::frt,*  *ΔzapA::zapA-mCherry-frt-cat-frt* | Peters *et al.* 2011 |  |
| JM144 | BW27783, *ΔftsN::frt-Ypet-ftsN, ΔzapA::zapA-mCherry-frt-cat-frt* | This study | P1(TU211) x STK9 |
| JM149 | BW27783  pDSW210-GFP | This study | BW27783 pDSW210-GFP |
| JM150 | BW27783, *ΔftsN::frt-Ypet-ftsN, ΔzapA::zapA-mCherry-frt-cat-frt*  pSEB306+ | This study | JM144 + pSEB306+ |
| JM151 | BW27783, *ΔftsN::frt-Ypet-ftsN, ΔzapA::zapA-mCherry-frt-cat-frt*  pSEB306+* | This study | JM144 + pSEB306+* |
| JM177 | BW27783,  *ΔzapA::zapA-mCherry-frt* | This study | P1(TU211) x BW27783; pCP20 |
| JM178 | BW27783,  *ΔzapA::zapA-mCherry-frt*  JW0093 | This study | JM177 + JW0093 |

**Table S2B.**  Plasmids used in this study.

| **Plasmids** | **Relevant genotype** | **Source** |
| --- | --- | --- |
| pDSW210 | ColE1 *ori*, P_206_ promoter, Amp^R^ | Pichoff & Lutkenhaus, 2005; Weiss *et al.* 1999 |
| pDSW210-GFP | GFP in pDSW210 | Lutkenhaus lab |
| pSEB306+ | *ftsA* in pDSW210 | Pichoff & Lutkenhaus, 2005 |
| pSEB306+* | *ftsA(R286W)* in pDSW210 | Pichoff & Lutkenhaus, 2005 |
| JW0093 | *ColE1 ori, PT5-lac::6xHis-ftsZ-gfp,* Cm^R^ | Yang et al., 2017 |
| pCP20 | *FLP recombinase expression;* Amp^R^ Cm^R^;  *temperature-sensitive replicon* | Cherepanov & Wackernagel 1995 |
